# Sodium Copper Chlorophyllin Immobilization onto *Hippospongia communis* Marine Demosponge Skeleton and Its Antibacterial Activity

**DOI:** 10.3390/ijms17101564

**Published:** 2016-09-27

**Authors:** Małgorzata Norman, Przemysław Bartczak, Jakub Zdarta, Wiktor Tomala, Barbara Żurańska, Anna Dobrowolska, Adam Piasecki, Katarzyna Czaczyk, Hermann Ehrlich, Teofil Jesionowski

**Affiliations:** 1 Institute of Chemical Technology and Engineering, Faculty of Chemical Technology, Poznan University of Technology, Berdychowo 4, 60965 Poznan, Poland; malgorzata.norman@hotmail.com (M.N.); przemyslaw.bartczak88@gmail.com (P.B.); jakub_zdarta@wp.pl (J.Z.); tomala.wiktor@gmail.com (W.T.); bzuranska@op.pl (B.Ż.); 2 Department of Biotechnology and Food Microbiology, Poznan University of Life Sciences, 60627 Poznan, Poland; anna.dobrowolska@up.poznan.pl (A.D.); kasiacz@up.poznan.pl (K.C.); 3 Institute of Materials Science and Engineering, Faculty of Mechanical Engineering and Management, Poznan University of Technology, Jana Pawla II 24, 60965 Poznan, Poland; adam.piasecki@put.poznan.pl; 4 Institute of Experimental Physics, TU Bergakademie Freiberg, Leipziger 23, 09599 Freiberg, Germany; hermann.ehrlich@physik.tu-freiberg.de

**Keywords:** marine sponge, *Hippospongia communis*, chlorophyllin, hybrid materials, antibacterial activity

## Abstract

In this study, *Hippospongia communis* marine demosponge skeleton was used as an adsorbent for sodium copper chlorophyllin (SCC). Obtained results indicate the high sorption capacity of this biomaterial with respect to SCC. Batch experiments were performed under different conditions and kinetic and isotherms properties were investigated. Acidic pH and the addition of sodium chloride increased SCC adsorption. The experimental data were well described by a pseudo-second order kinetic model. Equilibrium adsorption isotherms were determined and the experimental data were analyzed using both Langmuir and Freundlich isotherms. The effectiveness of the process was confirmed by ^13^C Cross Polarization Magic Angle Spinning Nuclear Magnetic Resonance (^13^C CP/MAS NMR), Fourier transform infrared spectroscopy (FTIR), energy-dispersive X-ray spectroscopy (EDS) and thermogravimetric analysis (TG). This novel SCC-sponge-based functional hybrid material was found to exhibit antimicrobial activity against the gram-positive bacterium *Staphylococcus aureus*.

## 1. Introduction

The utilization of materials of natural origin like both structural polysaccharides (chitin, cellulose) and structural proteins (collagen, keratin, silk, spongin) has been gaining increasing scientific attention in recent years. Key features contributing to the popularity of these renewable biomaterials include biodegradability, ecological safety, low cost, renewability, and high compatibility with the environment.

**Figure 1:**
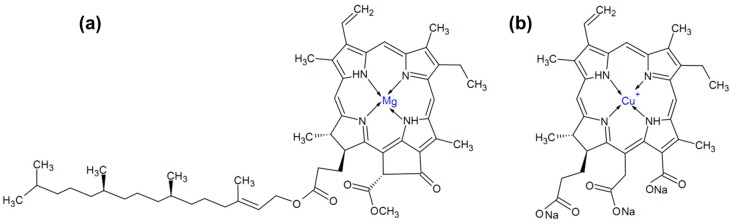
Chemical structure of chlorophyll (**a**) and chlorophyllin (**b**).

Chlorophyllin (see Figure 1b), a chlorophyll derivative, is obtained as a product of solvent extraction of grass, lucerne, nettle and other plant material. Further, saponification removes the methyl and phytol from the chlorophyll molecule, and may partially cleave the pentenyl ring (depending on the degree of hydrolysis [1], the cyclopentenyl ring may be cleaved with the resultant production of a third carboxyl function). This procedure leads to a complex mixture of compounds [2,3,4]. In chemical terms, this is a macrocyclic molecule consisting of four pyrrole rings connected by methylene bridges, with a metal ion inside (Figure 1). The most common form is the sodium–copper derivative (chlorophyllin sodium copper salt or sodium copper chlorophyllin–SCC), C_34_H_31_CuN_4_Na_3_O_6_ [5]. The replacement of the central magnesium ion with copper produces a more stable complex with greater tinctorial strength. As well as copper, divalent cations such as iron and zinc may be used [6,7,8,9].

Unlike chlorophyll, SCC is water soluble, due to replacement of the phytol with a carboxylate group [10]. Moreover, SCC is very slightly soluble in lower alcohols, ketones and diethyl ether. However, it is insoluble in chloroalkanes, hydrocarbons and fixed oils [3]. Chlorophyllins have great potential for biomedical application. SCC, considered as nontoxic, has been used historically in the treatment of several human conditions [11,12]. Nevertheless, more recent studies have shown tumor-enhancing and genotoxic effects of the complex [13]. Chlorophyllin exhibits antioxidant, antimutagenic, and anticarcinogenic properties in several models. It is known to bind to planar compounds such as heterocyclic amines [14,15], dioxin [16], aflatoxin [17,18], and benzo[*a*]pyrene [19]. The antimutagenic activity of chlorophyllin comes also from the scavenging of free radicals and active oxygen species, and suppression of or interference with metabolic activation by a specific cytochrome (P-450) and other metabolizing enzymes [20,21]. Other bioactivities are also attributed to chlorophyllin, such as immunomodulatory and antiapoptotic effects [22], antioxidant activity against oxidative stress or radiation-generated reactive oxygen species [23,24], and antibacterial effects [5,25,26,27]. SCC is used as a dietary supplement, in food, drugs and cosmetics [28], in textile dye [1,29], as an internal deodorizer [30] and as a natural wound healer [31].

There are few reports to date concerning the adsorption and further use of material composed of SCC with a support. Chlorophyllin with TiO_2_ is used as a photosensitizer [32] in artificial photosynthesis, chlorophyllin-chitosan as a trap for polycyclic mutagenic compounds [33], and heterocyclic amines to prevent their mutagenic action [14]. Copper chlorophyllin with hydrotalcite [34] and graphene oxide nanostructures [35] exhibits a bactericidal effect. SCC adsorbed onto silk can serve as natural dye for fibers [1]. It is also worth mentioning that copolymer of chlorophyllin sodium copper salt, acrylic acid, n-butyl acrylate, and N-isopropylacrylamide is used as light sensitive cation exchanger for lysozyme purification [36].

Due to their hierarchical, anastomosing structure, spongin-based skeletons of diverse spicule-free marine keratose sponges, also known as bath sponges, represent promising biological materials for use in several branches of science, biomedicine and technology. These demosponges are widely cultivated under marine ranching conditions and, consequently, represent renewable source of special, naturally prestructured biological scaffolds. Nevertheless, to date they have found application mostly as adsorbents [37,38], scaffolds for tissue engineering and regeneration [39,40,41,42] and templates for development of composites used in electrochemistry [43]. Therefore, it is necessary to further functionalize selected marine demosponge skeletons as special matrices in order to improve their surface properties and enable their use in various further applications. According to our opinion, the combination of chlorophyllin with a support consisting of marine demosponge spongin-based skeletons makes it possible to obtain a product which combines the desirable properties of both substrates: mechanical rigidity, high chemical and thermal resistance, biocompatibility and antibacterial properties. Our study focuses on obtaining novel, functionalized dye-biopolymer hybrid material which can holds great promise for applications in cosmetics, medicine and pharmacy (as a drug carrier or wound dressing).

## 2. Results and Discussion

### 2.1. Adsorption Tests

#### 2.1.1. Effect of Contact Time

**Figure 2:**
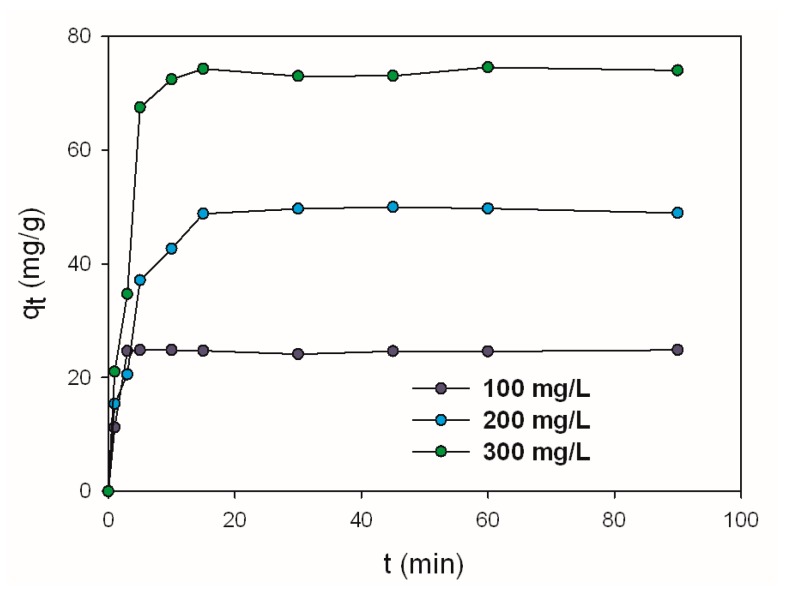
Effect of contact time and initial sodium copper chlorophyllin (SCC) concentration on the adsorption capacity of *H. communis* marine sponge skeleton (0.1 M NaCl).

Figure 2 shows the quantity of dye (SCC) adsorbed on the surface of the selected fragments of *H. communis* marine sponge skeletons as a function of time. The adsorption process was carried out for chlorophyllin solutions at concentrations of 100, 200 and 300 mg/L in the presence of 0.1 M NaCl, in a neutral environment, for a time of 1–90 min.

The results show that in the initial stage of the process there was a rapid rise in the quantity of dyes adsorbed (*q_t_*) for each concentration analyzed. With an increase in the initial dye concentration, the quantity adsorbed also increases. Regardless of the initial dye concentration, the adsorption efficiency is close to 100%. This demonstrates the high sorption capacity of *H. communis* skeletons with respect to chlorophyllin. After a certain time (approximately 30 min of the duration of the experiment) the value stabilizes. Our results for the efficiency of adsorption of SCC are better than others previously reported for different supports, for example, 49.75 g/kg SCC on silk fibers [1], and 5 μmol/10 mg SCC on chitosan [33].

It was also investigated how chlorophyllin adsorption on *H. communis* sponge skeletons varies as a function of the pH of the solution and of ionic strength. The results are presented in Table 1.

#### 2.1.2. Effect of Initial Dye Concentration

The above results indicate noticeable variation in the values of *q_t_* and *E*. It was found that, independently of the other process parameters, the quantity of the dye adsorbed increases with increasing initial concentration of the dye solution. This pattern is related to the quantity of dye molecules adsorbed on the surface of the organic support. The higher the dye concentration, the larger the number of particles present in the solution that can become bound to the adsorbent. On the other hand, an increase in concentration leads to saturation of the active sites on the support, as a result of which a significant quantity of dye particles are not adsorbed, causing a reduction in the process efficiency.

#### 2.1.3. Effect of pH

There are several factors that affect the stability of chlorophyll and chlorophyllin. As far as pH is concerned, all chlorophylls are most stable under alkaline conditions [44]. Analysis of the effect of pH showed that the type of environment plays a significant role in the process of adsorption of chlorophyllin on marine sponge skeletons studied. The quantity of dye adsorbed is greatest at pH = 5; for an initial concentration of 200 mg/L that value is 49.35 mg/g (without NaCl). By comparison, the *q_t_* values obtained in the same process conditions at pH = 7 (neutral pH) and pH = 11 are 6.82 and 5.60 mg/g, respectively. The process efficiency also indicates that an acidic environment is the best for adsorption (nevertheless, at pH = 3 chlorophylls will hydrolyze and lose color rapidly, and copper chlorophyllins will precipitate). In a neutral environment, there is large variation between the efficiency values obtained using solutions with concentrations ranging from 25 to 200 mg/L—the values for these extreme points are respectively 89.24% and 13.64%. For pH = 11, however, the values lie in the range of 3.95%–11.20%, while for pH = 5 they are close or equal to 100% (without NaCl). It can be seen that the decisive parameter is the acidity of the environment, not the initial dye concentration. The reason for this may be that in a low level pH environment the –NH_2_ groups of the proteinaceous matrix undergoes protonation to –NH_3_^+^. In this case, it is possible for the cationic groups to be substituted by negatively charged ions of chlorophyllin. Thus, the adsorption takes place by way of electrostatic interactions.

#### 2.1.4. Effect of Ionic Strength

The effect of ionic strength is visible only when a larger quantity of NaCl (0.1 M) has been used. In an acidic or alkaline environment, there is a minimal increase in the quantity of dye adsorbed on the support as the ionic strength increases, since the decisive factor is the pH. However, a clear effect can be seen in a neutral environment. When the process is carried out with a dye solution with a concentration of 200 mg/L to which no NaCl has been added, the value of *q_t_* is 6.82 mg/g, but when 0.1 M NaCl is added the quantity of dye adsorbed rises to 49.73 mg/g. Similarly, an increase in the efficiency of the process can be observed. This behavior may be caused by several factors. According to the surface chemistry theory, repulsion between the adsorbed molecules and non-adsorbed molecules in the solution is opposite to the adsorption process, especially when the surface concentration is high. The presence of additional ions from salt in the medium decreases the repulsion between adjacent dye particles, allowing the adsorbed molecules on the surface to be closer to each other. In addition, the electric double layer, which surrounded both adsorbent and adsorbate, is compressed at high ionic strength, resulting in lowering or elimination of the repulsive energy barrier: thus, the van der Waals forces become significant, leading to an increase in sorption of the dye on the particle surface [45,46]. This tendency is caused by a decrease in the repulsive electrostatic forces between the dye and the demosponge skeletons, causing an increase in adsorption when the ionic strength is increased [33].

### 2.2. Kinetic and Isothermal Studies

To determine the kinetics of the adsorption process, pseudo-first order (PFO) and pseudo-second order (PSO) models were used. These two models basically include all steps in the process, such as external film diffusion, adsorption, and internal particle diffusion, so they are pseudo-models [47]. This analysis makes it possible to describe the dependence of the adsorption process on time.

**Figure 3:**
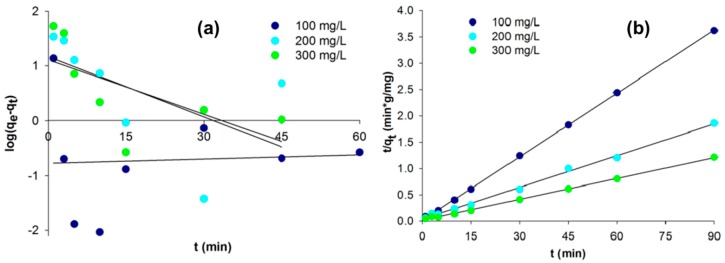
Plots of the pseudo-first order (**a**) and pseudo-second order (**b**) models at different initial dye concentrations.

The linearized integral form of the pseudo-first order model is generally expressed as:

(1) $$\log\left(q_{e}-q_{t}\right)=\log\left(q_{e}\right)-\frac{k_{1}}{2.303}\cdot t$$

where *q_t_* and *q_e_* are the adsorption capacities at time *t* and at equilibrium respectively (mg/g), *k*_1_ is the rate constant of pseudo-first order adsorption (min^−1^), and *t* is the contact time (min). Plotting log (*q_e_* − *q_t_*) versus *t* gives a linear relationship from which *k*_1_ and the predicted *q_e_* can be determined from the slope and intercept of the plot, respectively (Figure 3).

The simplified and linearized form of the pseudo-second order model is:

(2) $$\frac{t}{q_{t}}=\frac{1}{k_{2}q_{e}^{2}}+\frac{1}{q_{e}}\cdot t$$

where *k*_2_ (g/(mg·min)) is the second order rate constant of adsorption. The values of *k*_2_ and equilibrium adsorption capacity *q_e_* were calculated from the intercept and slope of the plot of *t*/*q_t_* versus *t*, according to Equation (2) and Figure 3 [48]. The kinetic process parameters are listed in Table 2.

The linear fit for *t*/*q_t_* vs. contact time and the *r^2^* value for the pseudo-second order kinetic model show that the dye adsorption kinetics can be approximated as pseudo-second order. Additionally, the experimental values *q_e,exp_* fit the calculated values *q_e,cal_* obtained from the linear plots of the pseudo-second order model better than the pseudo-first order values. In the case of the adsorption rate coefficient determined using the PSO model, a decrease can be observed as the dye concentration increases. This is due to an increase in the adsorbate’s competitiveness for the active sites capable of adsorbing it. For the coefficient *h* (initial adsorption rate) no dependency was found; this results from the heterogeneity of the material used.

The most popular isothermal theory for the adsorption of dyes onto biopolymers is the Langmuir model [49]. The Freundlich equation is also frequently applied in liquid–solid systems. According to this theory, dye concentrations on the adsorbent will increase so long as there is an increase in the dye concentration in the liquid. A basic assumption of the Langmuir theory is that sorption takes place at specific sites within the adsorbent, and therefore constitutes monolayer adsorption. Adsorption equilibrium is established when an adsorbate-containing phase has been in contact with the adsorbent for sufficient time, with the adsorbate concentration in the bulk solution in dynamic equilibrium with the interface concentration [50]. Based on experimental data, adsorption isotherms were plotted using the Freundlich and Langmuir models. The correlation of the experimental adsorption data with a number of adsorption models was investigated to gain an understanding of the adsorption behavior.

The nonlinear forms of the Freundlich (3) and Langmuir (4) equations are presented below:

(3) $$q_{e}=K_{f}\cdot C_{e}^{\frac{1}{n}}$$

where *C_e_* denotes the equilibrium concentration of the dye solution (mg/L), *q_e_* is the quantity of dye adsorbed at equilibrium (mg/g), and *K_f_* (mg/g) and *n* are the Freundlich constants. The values of *K_f_* and *n* can be determined from the intercept and gradient of the plot of log(*q_e_*) against log(*C_e_*).

(4) $$q_{e}=\frac{q_{m}\cdot b\cdot C_{e}}{1+b\cdot C_{e}}$$

where *C_e_* is the equilibrium concentration of the dye solution (mg/L), *q_m_* is the maximum adsorption capacity, and *b* is the Langmuir constant (L/mg), which is calculated from the intercept and downward linear slope of the graphs of *C_e_*/*q_e_* and *C_e_*.

**Figure 4:**
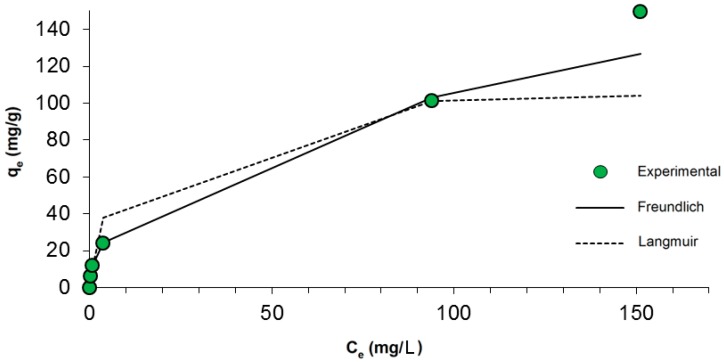
Fit of experimental data to the Langmuir and Freundlich models.

A graph of *q_e_* against *C_e_* for the adsorption isotherms of chlorophyllin on marine sponge skeletons is shown in Figure 4.

A value *n* < 1 or *n* > 1 implies that the adsorption process involves a chemical or favorable physical process, respectively. The *n* value here is 2.27, indicating a favorable process of adsorption of SCC on marine sponge skeletons. The Freundlich isotherm model fits the experimental data slightly better than the Langmuir model (correlation coefficient *R*^2^ = 0.995, compared with *R*^2^ = 0.992 for the Langmuir model). Maximum adsorption capacity, *q_m_*, is relatively high and equal to 108.56 mg/g, whereas *K_f_*, which is a Freundlich constant related to adsorption capacity, is equal to 13.97 mg/g. The isothermal parameters do not point clearly to either the Freundlich or the Langmuir equation, which suggests a combined sorption mechanism.

### 2.3. Desorption Test

First, adsorption of SCC onto *H. communis* sponge skeletons was carried out (30 min; pH = 7; 0.1 M NaCl; four different concentrations: 25, 50, 100, 200 mg/L). The adsorption efficiency and dye concentration in the adsorbent phase were calculated. Next, desorption of the previously obtained samples was carried out. The process conditions were the same as for adsorption, except that water was used in place of the dye solution with 0.1 M NaCl. The process efficiency was then calculated. The results indicate that the dye did not undergo desorption in the case of samples with initial concentrations of 25, 50, 100 and 200 mg/L. This suggests that the adsorption process is of a chemical nature, and that the chlorophyllin was deposited permanently on the spongin-based sponge skeletons.

### 2.4. Structural Analysis

**Figure 5:**
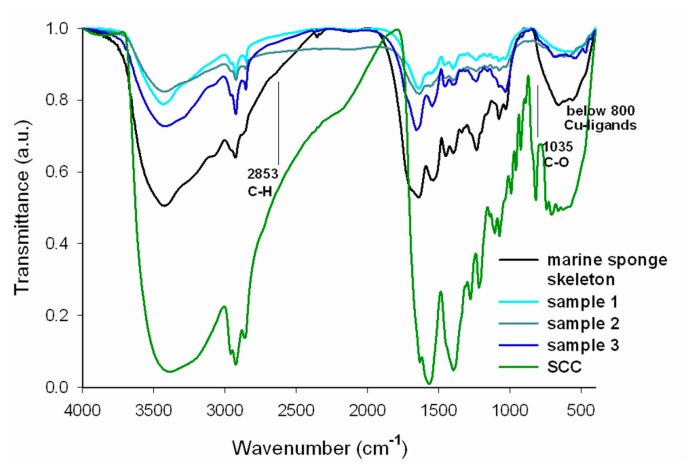
FTIR spectra of SCC, *H. communis* sponge skeletons and selected samples (sample 1: 50 mg/L, sample 2: 100 mg/L, sample 3: 200 mg/L; 60 min; pH neutral; 0.1 M NaCl).

The results of FTIR analysis of SCC, *H. communis* sponge skeletons and selected samples are presented in Figure 5.

In the SCC spectrum (green line), absorption bands are observed in the range of 3600–2850 cm^−1^ attributable to stretching vibrations of O–H, N–H and C–H bonds. The signal with maximum intensity at 3405 cm^−1^ is assigned to the stretching vibrations of OH groups in water molecules, while the bands at wavenumbers 2929 and 2859 cm^−1^ are related to symmetric and asymmetric stretching vibrations of C–H bonds, and that at 1632 cm^−1^ stretching vibrations of the carboxylate anion. The signals appearing in the range 1600–1300 cm^−1^ probably result from skeletal vibrations of macrocyclic ring of tetrapyrrole or the alkyl substituents in the porphyrin ring. The chlorophyllin spectrum contains a band at 1565 cm^−1^, which can be attributed to C=C and C=N bonds (skeletal vibrations of the porphyrin ring). The signals below 1000 cm^−1^ generally arise from molecular motion of carbon atoms, and the peaks at 991, 959 and 924 cm^−1^ can be attributed to the C–C stretching and bending vibrations of the pyrrole ring. The peak at 711 cm^−1^ results from vibrations of the metal atom (copper) present in the structure of the analyzed porphyrin. All of the assignments were made in accordance with [21,51,52].

On the FTIR spectrum for the spongin (black line), the wide band at 3500–3200 cm^−1^ is characteristic for deforming stretching vibrations of the hydroxyl group. Vibrations related to the presence of that group are also responsible for the signals at wavenumbers 1390 and 660 cm^−1^. The signal at 2930 cm^−1^ is attributed to stretching vibrations of C–H bonds, and that at 1390 cm^−1^ to deformation vibrations of the methyl group –CH_3_. The band at 1640 cm^−1^ indicates the presence of stretching vibrations of the carbonyl chromophore C=O. The presence of aromatic rings in the structure of the spongin is confirmed by the two bands at 1540 and 1450 cm^−1^, both of which are attributed to vibrations of coupled C=C bonds in the aromatic ring. The signal at 1230 cm^−1^ corresponds to stretching vibrations of C–O bonds in carboxyl groups. The remaining two bands at 1070 and 1020 cm^−1^ are characteristic for stretching vibrations of C–O bonds in alcohols.

The FTIR spectrum of the *H. communis* sponge skeleton fragments studied with adsorbed chlorophyllin (cyan, dark cyan and dark blue lines) contains signals originating both from the support and from the dye. The wide bands in the range 3500–3200 cm^−1^ are characteristic for stretching vibrations of OH groups. The increase in the intensity of the signals at wavenumbers 2927 and 2853 cm^−1^, reflecting the presence of C–H bonds, and of the signal at 1659 cm^−1^ assigned to C=O bonds, proves the effective adsorption of the dye onto the sponge skeleton. There are also bands at 1548, 1459 and 1403 cm^−1^, which can be attributed to skeletal vibrations of the porphyrin ring formed by C=C and C=N bonds (from SCC). The peaks at 1239 and 1035 cm^−1^ indicate stretching vibrations of C–O (their source is in marine sponge skeleton), while the signals with maxima at 916 and 801 cm^−1^ can be assigned to vibrations of C–C bonds in the pyrrole ring. The signals in the wavenumber range 700–550 cm^−1^ are generated by bonds formed between copper ions and chlorophyllin, providing further confirmation of the successful immobilization of the dye.

It should also be noted that the small shifts in the maximum wavenumbers of certain peaks (OH and NH groups, C=O bonds) may suggest that the interactions between SCC and *H. communis* sponge skeletons are generally based on ionic interactions. This observation is in agreement with the observations concerning changes in process efficiency depending on changes in the pH of the solution.

The results of energy-dispersive X-ray spectroscopy (EDS) are given in Table 3.

The marine sponge skeleton studied here is composed mainly of carbon, nitrogen and oxygen. These elements make up a protein structure (spongin), which is the main component of the skeleton of *H. communis*. EDS analysis also reveals the presence of chlorine, aluminum, silicon, sulfur and iodine (in the form of 3,5-diiodotyrosine, called iodogorgonic acid) [53]. It has been proved that halogens exist in combination with organic components in demosponges [54]. The data from EDS analysis confirm indirectly the effectiveness of adsorption process, elements both from SCC and *H. communis* skeleton are presented in obtained hybrid material.

**Figure 6:**
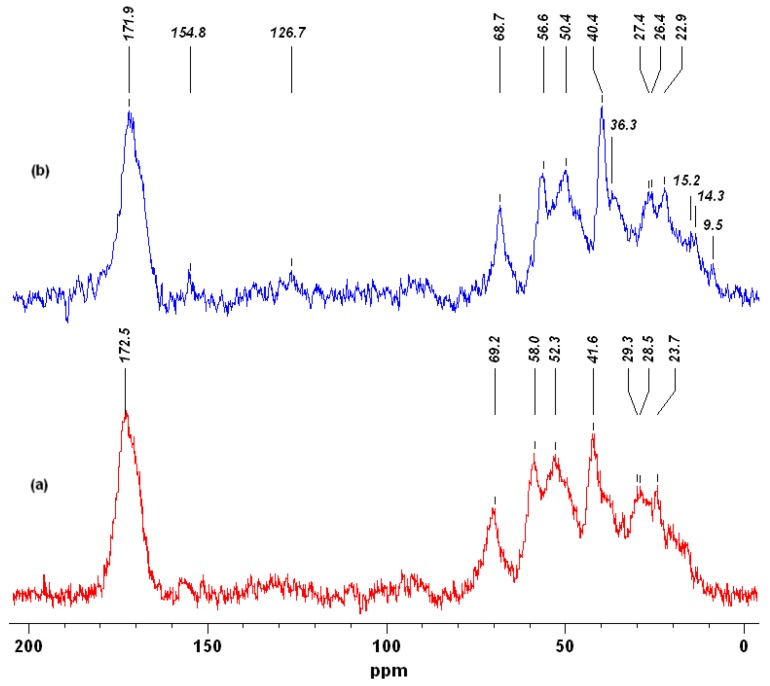
^13^C CP/MAS NMR spectra of *H. communis* sponge skeletons (**a**) and developed hybrid material (**b**) (750 mg/L; 30 min; pH neutral; 0.1 M NaCl) (additional signals are written horizontally).

The ^13^C CP/MAS NMR spectra of *H. communis* sponge skeletons and selected hybrid materials (obtained from 750 mg/L SCC, which give 101.5 mg of dye on 1 g of *H. communis* skeleton) are presented in Figure 6. The additional signals (in comparison with (a), written horizontally) visible on spectrum (b) come from the SCC adsorbed on the sponge skeletons studied. Based on previously published data [55,56] they can be assigned to: C1, C6 or C16 carbons from the porphyrin macrocycle (154.8 ppm); C13 (126.7 ppm) carbon chains with double bond; C17 and C17′ from the reduced phytol chain (36.3 ppm); C8′, C8′′(15.2; 14.3 ppm), C2′, C7′, C12′ (9.5 ppm) from the macrocycle’s short carbon chain substituent. Moreover, adsorption of the dye induces slight differences in the values of the signals which are present in the spectra of both the investigated marine sponge skeletons and the hybrid material developed.

Data from spectroscopic analysis provides important information both about *H. communis* structure and possible mechanism of interaction between SCC and spongin-based sponge skeletons. Hydrogen bonds formation and electrostatic interaction play a key role in this process. Indirect confirmation of this assumptions is also provided by results from adsorption studies.

**Figure 7:**
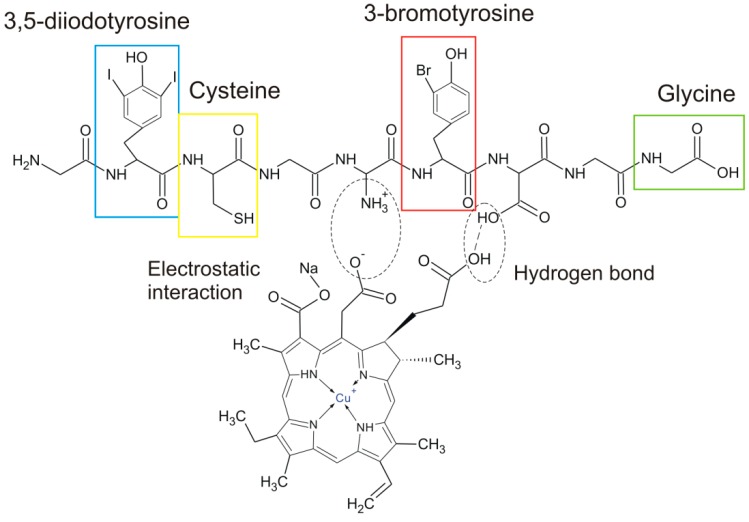
Proposed mechanism of interaction between SCC and spongin-based sponge skeletons (typical spongin-build amino acids are marked in frames).

Figure 7 presents the illustrative combination of typical amino acids, which build the sponging structure as well as possible interaction between the functional group of these amino acid and SCC molecule.

**Figure 8:**
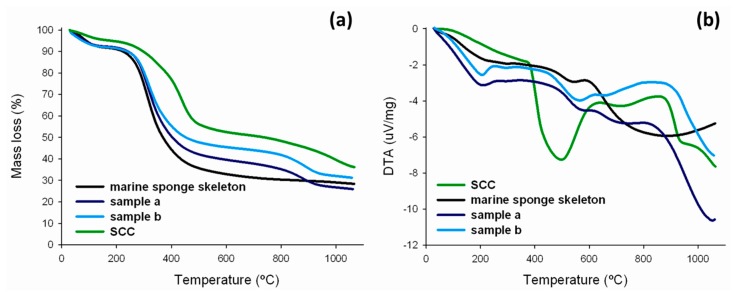
TG (**a**) and DTA (**b**) curves for *H. communis* sponge skeletons, SCC, and selected samples (sample a: 400 mg/L, sampleb: 750 mg/L; 30 min; pH neutral; 0.1 M NaCl).

Sodium copper chlorophyllins are relatively thermally stable [51]. As shown in Figure 8a, the thermogram of SCC contains three discrete characteristic decomposition steps. The first weight loss, of about 4%, between 85 and 115 °C can simply be ascribed to the escape of moisture or air adsorbed in the sample [57]. According to [51], the second decomposition step, in the temperature range 240–370 °C, with a percentage loss of 20%, may be attributed to the elimination of Cu^2+^ ions. The maximum rate of mass loss (about 40%) of SCC occurred at 390–510 °C and matches an observed exothermic peak (Figure 8b), which is indicative of the rupture and degradation of the porphyrin macrocycle [58]. From a temperature of 700 °C, there is another small drop in mass (to 60%), which may be associated with combustion of the organic matrix. Nevertheless, SCC, like the sponge skeletons, does not show complete loss of mass in TG-DTA measurements, because of the coal formation [58].

The thermogram of the *H. communis* sponge skeletons have been recently described in detail in [38]. The thermogram of the spongin-based skeleton studied in this work with adsorbed chlorophyllin shows three characteristic decomposition stages. The first mass loss, of around 10%, observed between 80 and 250 °C, is associated with evaporation of the residual water in the sample. The second stage occurs in the temperature range 300–600 °C, with a 40% mass loss, which may be attributed to the elimination of Cu^2+^ ions or to thermal decomposition of the organic phase. Another small mass loss (of around 10%) occurs in the range 800–1000 °C, this may reflect combustion of the organic matrix. The thermogram shows that when the dye is applied to the *H. communis* sponge skeletons their thermal stability increases, and the greater the quantity of adsorbed chlorophyllin, the greater the resistance to thermal decomposition.

Using an optical microscope and a scanning electron microscope (SEM), photographs were taken to enable precise analysis of the morphology and microstructure of the sponge skeleton before and after the adsorption process.

**Figure 9:**
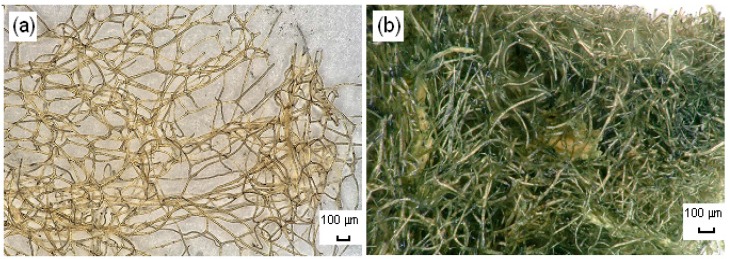
Light microscopy images of *H. communis* sponge skeletons before (**a**) and after (**b**) adsorption of SCC.

**Figure 10:**
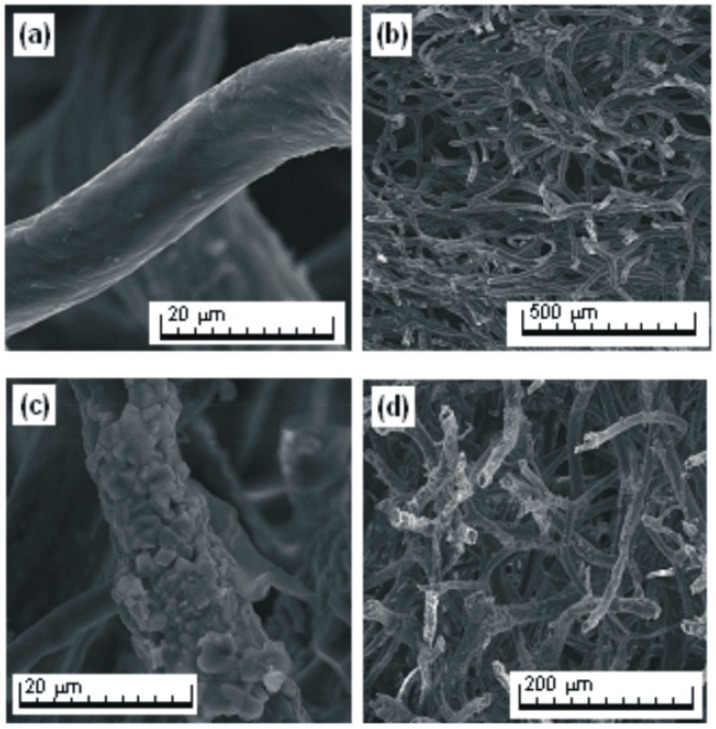
SEM images of purified *H. communis* sponge skeletons before (**a**,**b**) and after (**c**,**d**) adsorption of SCC, at different magnifications.

The photographs of the spongin-based skeleton fragments (Figure 9a and Figure 10a,b) indicate their typical fibrous, reticulate structure. Single fibers, composed of microfibers, combine into a complex hierarchical network. Subsequent photographs (Figure 10c,d) show that the fibers are covered by a layer of the adsorbed dye (750 mg/L, 30 min, pH neutral, 0.1 M NaCl).

### 2.5. Antibacterial Tests

The results of antibacterial activity for the analyzed materials against *Staphylococcus aureus* are presented and described below. Tetracycline antibiotic was used as a positive control.

The results demonstrate that the *H. communis* sponge skeleton–SCC hybrid material reduced the growth of the above-mentioned gram-positive bacteria. Although the effect is not observed for the first two samples (0.1 and 1 mg), antibacterial activity emerged and increased with higher contents of SCC (for 5 and 10 mg of weighted portion of hybrid material the inhibition zone diameter is equal to 13 and 14 mm, respectively). The absence of antibacterial activity does not imply the absence of bioactive components, but active constituents may be present in insufficient quantities to inhibit cell growth. Lack of activity can thus only be proven by using large doses [59]. According to [60], the diameter of the halo zone indicates that the hybrid material exhibits good antibacterial activity. Also, an extract from this sponge species does not exhibit antibacterial properties against *Straphylococcus aureus*, as reported in [61]. In comparison, 30 μg of Tetracycline reduce the growth of *S. aureus* less that examined hybrid material, the halo zone is 7 mm. *H. communis* sponge skeletons without any modification showed no antibacterial activity against the tested bacterial species. The hybrid of SCC and *H. communis* sponge skeleton is not so active as SCC (as results from conversion of the actual quantities of SCC in 100 μL of solution and 100 μL of suspension), inhibition zone diameter is equal to 12 and 14 mm, for 100 mL of 100 and 400 mg/L of sodium copper chlorophyllin solution, respectively. Nevertheless, there are other factors which make this material much more useful than the pure dye, for example, its insolubility provides the possibility of reusing it. The same behavior was also reported previously [35]. The antibacterial activity of SCC can be explained by its excellent photosensitizing properties. When the molecules are activated by visible light, they generate singlet oxygen, which is cytotoxic to most living cells. When these short-life free radicals are close enough to the cell surface, they can trigger extensive disruption in the cell membrane, resulting in cell death [5].

Moreover, similar experiments were performed against gram-negative bacteria *Escherichia coli* and *Pseudomonas aeruginosa*, using both SCC alone and obtained hybrid material, but any antimicrobial activity was observed (data not provided). Different activity of SCC against gram-positive and gram-negative bacteria could be explained by different cell membrane structures. In both cases, the cell wall is constructed from the polymer peptidoglycan, which is thicker in gram-positive bacteria (*S. aureus*). In contrast, gram-negative bacteria (*E. coli* and *P. aeruginosa*) have a thin layer of peptidoglycan, but it is located between the cytoplasmic membrane and an extra, second membrane called the outer membrane made of phospholipids and lipopolysaccharides [62,63], which probably made them resistant to SCC activity.

## 3. Materials and Methods

### 3.1. Materials

Marine demosponges of the species *Hippospongia communis*, purchased from INTIB GmbH (Freiberg, Germany), were originally collected in the Mediterranean Sea (Tunisia). To ensure the uniformity, all the samples were prepared according to the same procedure. The sponge material was washed with distilled water to remove salts. After that, it was stored for 3 days in 3 M HCl at room temperature with the aim to dissolve possible calcium carbonate-containing contaminations. The material was then rinsed with distilled water up to pH 6.5 and dried during 24 h at 50 °C.

Chlorophyllin sodium copper (SCC) salt was from Sigma Chemical Co. (St. Louis, MO, USA) and used as supplied. The stock solution was prepared by dissolving an appropriate amount of dye in 1 L of distilled water. Experimental solutions of desired concentrations were obtained by successive dilutions with distilled water. Other chemicals were of reagent grade and used without further purification.

### 3.2. Adsorption and Desorption Tests

Batch experiments were performed to investigate the effect of contact time and to determine kinetic parameters. Adsorption experiments were performed using glass bottles containing appropriate quantities of marine sponge skeleton and dye solution. The initial concentration of the dye was 100, 200 and 300 mg/L. SCC was dissolved in water.

After different time intervals, the samples were filtered off under vacuum and analyzed using a UV-VIS spectrophotometer (Jasco V750, Jasco, Tokyo, Japan) at maximum absorbance wavelength 405 nm. After filtration each sample was left to dry in room temperature for 48 h. Dye concentration in the adsorbent phase at a specific time (*q_t_*) (5) and the adsorption efficiency (*E*) (6) were calculated as:

(5) $$q_{t}=\frac{\left(C_{0}-C_{t}\right)\cdot V}{m}$$

(6) $$E\left(\%\right)=\frac{C_{0}-C_{t}}{C_{0}}\cdot 100\%$$

where *C*_0_ and *C_t_* are the concentrations of the dye in the solution before and after sorption, respectively (mg/L), *V* denotes the volume of the solution (L), and *m* is the mass of sorbent (g).

The effect of pH on the adsorption of SCC from aqueous solution by selected fragments of the marine sponge skeleton was similarly investigated. The pH was adjusted to 3, 5, 7, 9 and 11 using either 0.1 M HCl or 0.1 M NaOH.

The effect of ionic strength on dye sorption was studied by adding NaCl (0.01 and 0.1 M) to 50 mL of SCC solution.

Adsorption isotherms were obtained by placing the samples of selected sponge skeletons in a series of flasks containing 50 mL of dye solution at different initial concentrations (25–1000 mg/L + 0.1 M NaCl) prepared from stock solutions. After shaking (30 min), the residual concentration of the dye was estimated.

The next step was the evaluation of the hybrid material’s stability (desorption tests). To a conical flask containing 50 mL water, a portion of the obtained hybrid material was added. The suspension was shaken for 4 h at room temperature. The suspension was filtered off under reduced pressure. In the filtrate, the concentration of the soluble components was evaluated by absorbance measurements, and the concentration of the eluted dye was determined from the calibration curve.

### 3.3. Analysis

The dye-biopolymer hybrid material and precursors (dye and marine sponge skeleton) were also subjected to FTIR spectral analysis (using a Vertex 70 spectrometer, Bruker, Billerica, MA, USA). The materials were analyzed in the form of tablets, made by pressing a mixture of 250 mg anhydrous KBr and 1 mg of the analyzed substance under a pressure of approximately 10 MPa. The investigation was performed over a wavenumber range of 4000–400 cm^−1^ (at a resolution of 0.5 cm^−1^).

The surface composition was analyzed using a Tescan scanning electron microscope equipped with a PTG Prism Si(Li) (Princeton Gamma Tech., Princeton, NJ, USA) energy-dispersive X-ray spectrometer (EDS). Before the analysis, samples were placed on the ground with a carbon paste or tape. The presence of carbon materials is needed to create a conductive layer which ensures the delivery of electric charge from the sample.

Cross Polarization Magic Angle Spinning Nuclear Magnetic Resonance (^13^C CP/MAS NMR) analysis was performed using a DSX spectrometer (Bruker, Billerica, MA, USA). For this purpose 100 mg of a sample was located in a rotator, made of ZrO_2_, 4 mm in diameter and centrifuged at a spinning frequency of 8 kHz. ^13^C CP/MAS NMR spectra were measured at 100.63 MHz in a standard 4 mm MAS probe using a single pulse excitation with high power proton decoupling (pulse repetition 10 s, spinning speed 8 kHz).

The heat effects were determined by thermogravimetric analysis (TG and DTA) on a thermoanalyzer (Jupiter STA 449F3, Netzsch, Selb, Germany). Samples (approximately 5 mg) were heated to 1000 °C from room temperature at a rate of 10 °C/min, in a nitrogen atmosphere.

Images were made by means of a Keyence VHX-5000 (Keyenece, Osaka, Japan) digital optical microscope. The microstructures were studied under a Tescan Vega 5135 scanning electron microscope, in order to obtain data on surface morphology and structure.

### 3.4. Antibacterial Tests

Tests were carried out on systems composed of a dye and *H. communis* sponge skeletons (obtained from a 1000 mg/L SCC solution). Weighed portions were suspended in 1 mL of sterile water. The control consisted of a tetracycline antibiotic (30 μg), SCC solutions (100 and 400 mg/L) and sponges without the addition of dyes. Suspensions of indicator microorganisms, secured and stored at −20 °C, were thawed at room temperature, and then transferred to test tubes containing 10 mL of stock medium with the addition of 2% glucose (Oxoid, Basingstoke, UK). Culture took place at 35 ± 2 °C for 24 h. The tested bacterial suspensions containing 10^6^ cfu/mL were spread on Mueller-Hinton Agar (Oxoid) and left for 15 min to absorb the microorganisms on the surfaces. The indicator microorganisms used in the test was *Staphylococcus aureus* species. At the next stage of testing, on the surface of the prepared medium, wells 9 mm in diameter were made, into which 100 μL of suspension (in the case of the weighed samples) or 100 μL of solution (in the case of SCC) was applied. The Petri dishes with the applied samples were then incubated at a temperature of 35 ± 2 °C for 24 h. The antibacterial activity was evaluated based on the diameter of the zone in which growth of the indicator bacteria was inhibited.

## 4. Conclusions

Marine sponge skeletons isolated from *Hippospongia communis* are suitable as a solid support for sodium copper chlorophyllin. SCC can be tightly fixed on the support surface. The results of analysis suggest electrostatic interaction and hydrogen bonding between these two constituents. Adsorption conditions, especially pH and addition of salt, have a considerable effect on the results obtained. The adsorption rate was very high in the initial period of contact, due to the availability of reactive sites on the adsorbent. After equilibrium, no significant changes in adsorption efficiency are observed, because of saturation of the adsorbent after a longer contact time. The kinetics of the adsorption process is best described by a pseudo-second order model. Isothermal parameters do not indicate clearly either a Freundlich or a Langmuir model, which suggests a complex sorption mechanism. The results of TG, ^13^C CP/MAS NMR and FTIR analysis indirectly confirm the effectiveness of the process, as does EDS analysis, which also supplies additional information concerning the structure of the marine sponges. The antibacterial activity of the hybrid made of SCC with marine demosponge skeleton—an environmentally friendly and cost-effective material—against *S. aureus* was investigated and proved for the first time.

## Figures and Tables

**Table 1:** Influence of pH and ionic strength on sodium copper chlorophyllin (SCC) adsorption capacity on *H. communis* spongin-based skeleton (contact time 60 min).

Dye Concentration (mg/L)	Ionic Strength (mol/L NaCl)	pH = 5		pH = 7		pH = 11	
		*q_t_* (mg/g)	*E* (%)	*q_t_* (mg/g)	*E* (%)	*q_t_* (mg/g)	*E* (%)
25	-	6.25	100	5.58	89.24	0.25	3.95
50		12.50	100	3.78	30.24	1.32	10.58
100		24.96	99.85	5.49	21.96	2.72	10.87
200		49.35	98.70	6.82	13.64	5.60	11.20
25	0.01	6.25	100	4.61	73.72	1.00	15.97
50		12.50	100	3.17	25.37	0.44	3.49
100		25.00	100	4.67	18.67	1.01	4.04
200		49.96	99.93	6.35	12.70	2.90	5.81
25	0.1	6.25	100	5.99	95.86	2.50	40.08
50		12.50	100	12.34	98.72	3.12	24.92
100		25.00	100	24.57	98.29	4.75	19.00
200		50.00	100	49.73	99.46	6.97	13.94

**Table 2:** Kinetic constants of SCC adsorption on *H. communis* sponge skeletons at different dye concentrations (100, 200 and 300 mg/L; pH = 7; 0.1 M NaCl).

Kinetic Models	Parameters	Initial Dye Concentration		
		100 (mg/L)	200 (mg/L)	300 (mg/L)
**Pseudo-First Order**	*q_e,exp_* (mg/g)	24.84	49.73	74.57
	*q_e,cal_* (mg/g)	0.17	14.62	12.80
	*k*_1_ (1/min)	0.006	0.084	0.076
	*r*^2^	0.003	0.327	0.404
**Pseudo-Second Order**	*q_e,cal_* (mg/g)	24.87	49.83	75.85
	*k*_2_ (g/mg·min)	0.102	0.010	0.009
	*r*^2^	0.999	0.997	0.999
	*h*	63.07	24.80	50.94

**Table 3:** EDS analysis results.

Element	Content of Elements by Weight (%)			
	Chlorophyllin	Sponge Skeleton (SS)	SS + SCC (400 mg/L)	SS + SCC (1000 mg/L)
C	84.66	81.67	88.13	82.85
N	4.48	3.18	0.20	2.56
O	4.84	10.37	2.62	8.08
Cu	2.80	-	0.26	0.53
Cl	0.42	0.34	5.45	1.52
Na	2.79	-	1.91	0.80
Al	-	1.17	0.27	0.77
Si	-	0.25	0.04	0.10
S	-	1.15	0.31	0.87
I	-	1.87	0.82	1.92
Total	100.00	100.00	100.00	100.00
